# Association between birth weight and neurodevelopmental disorders assessed using the Korean National Health Insurance Service claims data

**DOI:** 10.1038/s41598-022-06094-x

**Published:** 2022-02-08

**Authors:** In Gyu Song, Han-Suk Kim, Yoon-Min Cho, You-na Lim, Duk-Soo Moon, Seung Han Shin, Ee-Kyung Kim, Joonsik Park, Jeong Eun Shin, Jungho Han, Ho Seon Eun

**Affiliations:** 1 Department of Pediatrics, Severance Children’s Hospital, Yonsei University College of Medicine, Seoul, South Korea; 2 Department of Pediatrics, Korea University Guro Hospital, Korea University College of Medicine, Seoul, South Korea; 3 Department of Pediatrics, Seoul National University Children’s Hospital, Seoul National University College of Medicine, 101, Daehak-ro, Jungno-gu, Seoul, 03080 Republic of Korea; 4 Health Insurance Research Institute, National Health Insurance Service, Wonju, South Korea; 5 Graduate School of Public Health, Seoul National University, Seoul, South Korea; 6 Department of Psychiatry, Jeju National University Hospital, Jeju, South Korea

**Keywords:** Psychiatric disorders, Medical research, Paediatric research

## Abstract

The risk of neurodevelopmental disorders in low birth weight (LBW) infants has gained recognition but remains debatable. We investigated the risk of attention-deficit/hyperactivity disorder (ADHD) and autism spectrum disorder (ASD) in school-aged children according to their birth weight. We conducted a retrospective cohort study using the Korean National Health Insurance claims data of 2,143,652 children who were born between 2008 and 2012. Gestational age of infants was not available; thus, outcomes were not adjusted with it. Not only infants with birth weights of < 1.5 kg, but also 2.0–2.4 kg and 1.5–1.9 kg were associated with having ADHD; odds ratio (OR), 1.41 (95% confidence interval [CI] 1.33–1.50), and 1.49 (95% CI 1.33–1.66), respectively. The OR in infants with birth weights of 2.0–2.4 kg and 1.5–1.9 kg was 1.91 (95% CI 1.79–2.05) and 3.25 (95% CI 2.95–3.59), respectively, indicating increased odds of having ASD. Subgroup analysis for children without perinatal diseases showed similar results. In this national cohort, infants with birth weights of < 2.5 kg were associated with ADHD and ASD, regardless of perinatal history. Children born with LBW need detailed clinical follow-up.

## Introduction

Birth weight is an important health indicator of optimal child health and development. Low birth weight (LBW, birth weight less than 2.5 kg) is often associated with a higher risk of neonatal mortality and increased utilization of healthcare services during the first year of life^1,2^. In the Republic of Korea (Korea), in 2019, the total fertility rate dropped to a low record of 0.92. However, the proportion of LBW infants has increased from 3.8 to 6.6% of the total live births in the last two decades. Importantly, those with birth weights between 1.5 and 2.4 kg accounted for 5.8% of the total live births in 2019^3^.

LBW infants may experience behavioral and emotional problems. Previous studies have revealed LBW to be associated with lower academic achievement, lower intelligence quotient, and developmental and behavioral disorders, such as attention-deficit/hyperactivity disorder (ADHD) and autism spectrum disorder (ASD)^4–6^. ADHD is one of the most common neurodevelopmental disorders in childhood. Children and adolescents with ADHD have inattention and/or hyperactivity-impulsivity patterns that affect functions and development. The estimated prevalence of ADHD ranged from 3.4% to 5.3% in children and adolescents worldwide^7,8^. ASD is characterized by persistent deficits in social communication and interaction and the presence of restricted and repetitive behaviors^9^. Additionally, ASD is associated with considerable lifetime costs not only to the individual and their family but also to the society^10^. Although the results of the meta-analyses indicated that LBW or preterm infants had an increased ADHD or ASD risk, most studies have focused on very low birth weight infants (VLBW, birth weight less than 1.5 kg) or very preterm infants (aged 28–31 gestational weeks)^5,6^. There are ongoing debates on the developmental and behavioral disorders of infants with birth weights between 1.5 and 2.4 kg or late preterm infants (aged 34–36 gestational weeks)^11,12^. Because of the increasing prevalence of LBW infants, it is necessary to evaluate the neurodevelopmental prognosis of these children.

This study aimed to address this knowledge gap by conducting a nationwide retrospective cohort study in Korea using the National Health Insurance Service (NHIS) claims data. We assessed the association between birth weight and two neurodevelopmental disorders (ADHD and ASD); especially, those whose birth weight ranged from 1.5 to 2.4 kg were investigated. We also aimed to evaluate the outcomes of these children according to their congenital or perinatal medical history.

## Results

**Figure 1:**
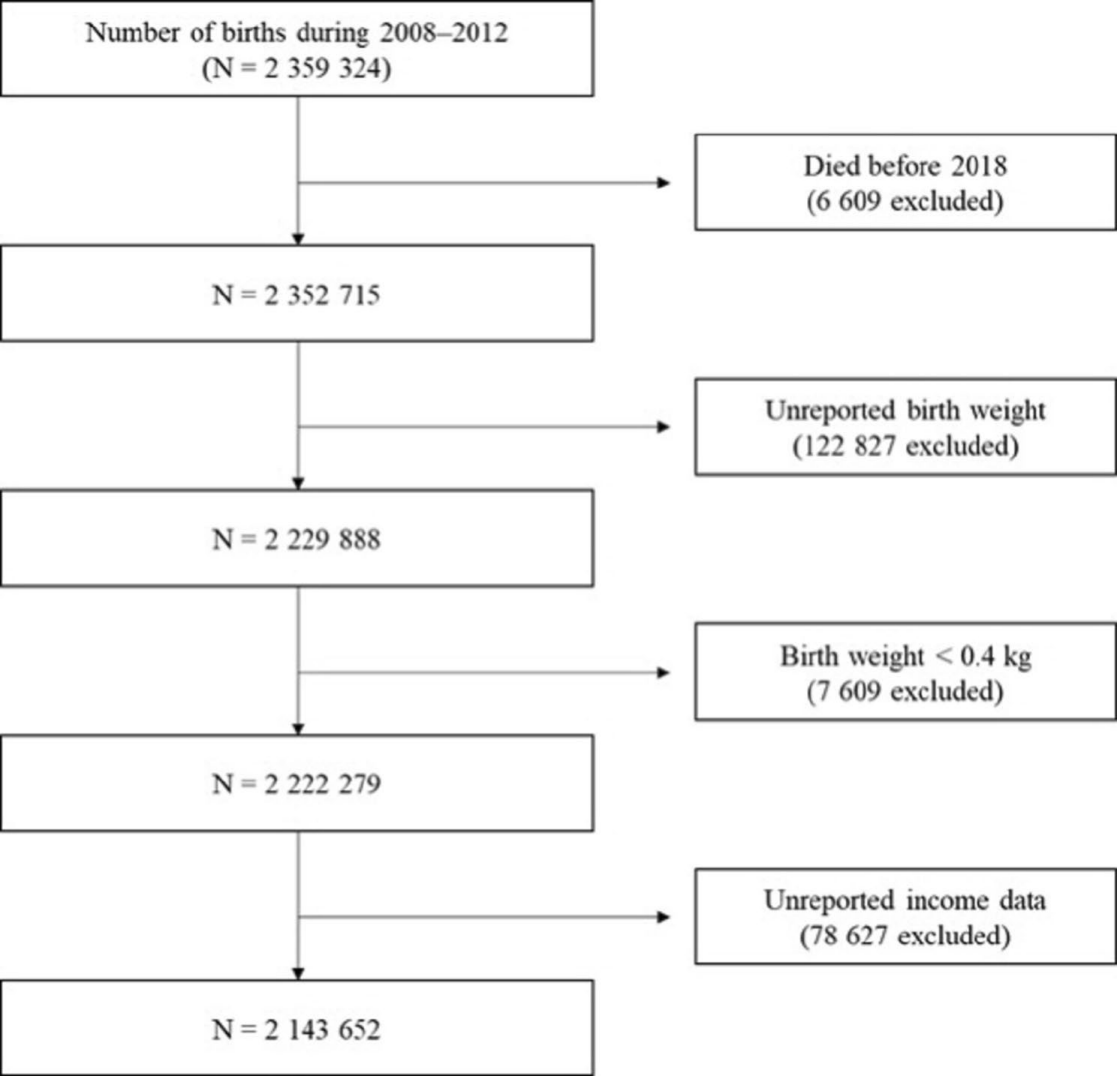
Flow chart of study participant selection.

A total of 2,359,324 live births were identified between 2008 and 2012 through the database in Korea. Among these data, children who died before 2018, had missing birth weight records, missing income data information, and had a birth weight of less than 0.4 kg were excluded. Thus, this study evaluated 2,143,652 live birth infants (Fig. 1, Supplementary Table S1). As we were unable to obtain gestational age of the infants, the results will be presented according to their birth weight.

### Demographics

Table 1 summarizes the demographic characteristics of infants at birth according to birth weight. The proportions of the medical aid group were larger in the LBW group than the normal birth weight (NBW) group. More than 80% of children with VLBW were diagnosed with congenital or perinatal diseases, while it was only 20.8% in with NBW.

**Table 1:** Demographics and proportion of children diagnosed with ADHD and ASD born between 2008 and 2012 in the Republic of Korea (n, %).

		Total	< 1.0 kg	1.0–1.4 kg	1.5–1.9 kg	2.0–2.4 kg	2.5–4.0 kg	> 4.0 kg	*P* value
Birth year^a^	2008	413,756 (100)	379 (0.1)	1666 (0.4)	3411 (0.8)	14,069 (3.4)	383,565 (92.7)	10,666 (2.6)	< 0.001
	2009	403,475 (100)	448 (0.1)	1637 (0.4)	3321 (0.8)	13,818 (3.4)	374,374 (92.8)	9877 (2.5)	
	2010	431,678 (100)	523 (0.1)	1651 (0.4)	3677 (0.9)	14,862 (3.4)	400,545 (92.8)	10,420 (2.4)	
	2011	438,828 (100)	557 (0.1)	1686 (0.4)	3706 (0.8)	15,960 (3.6)	407,290 (92.8)	9629 (2.2)	
	2012	455,915 (100)	605 (0.1)	1761 (0.4)	4105 (0.9)	16,918 (3.7)	422,279 (92.6)	10,247 (2.3)	
Sex	Female	1,040,430 (48.5)	1396 (55.6)	4204 (50.0)	9206 (50.5)	41,134 (54.4)	966,645 (48.6)	17,845 (35.1)	< 0.001
Income	Medical aid	11,872 (0.6)	20 (0.8)	69 (0.8)	138 (0.8)	580 (0.8)	10,700 (0.5)	365 (0.7)	< 0.001
	1st	233,822 (10.9)	276 (11.0)	942 (11.2)	2121 (11.6)	8593 (11.4)	215,988 (10.9)	5902 (11.6)	
	2nd	488,349 (22.8)	540 (21.5)	1941 (23.1)	4123 (22.6)	17,342 (22.9)	452,290 (22.8)	12,113 (23.8)	
	3rd	842,080 (39.3)	1,034 (41.2)	3250 (38.7)	6850 (37.6)	28,934 (38.3)	782,452 (39.4)	19,560 (38.5)	
	4th (highest)	567,529 (26.5)	642 (25.6)	2199 (26.2)	4988 (27.4)	20,178 (26.7)	526,623 (26.5)	12,899 (25.4)	
Congenital or perinatal diseases	Yes	479,204 (22.4)	2276 (90.6)	6838 (81.4)	13,476 (73.9)	30,795 (40.7)	413,657 (20.8)	12,162 (23.9)	< 0.001
ADHD^b^	Yes	27,008 (2.2)	63 (4.7)	186 (3.8)	346 (3.3)	1219 (2.9)	24,412 (2.1)	782 (2.5)	< 0.001
	Diagnosis age [Mean (S.D.)]	7.1 (1.6)	7.0 (1.8)	7.2 (1.6)	7.0 (1.6)	7.0 (1.6)	7.1 (1.6)	7.1 (1.6)	0.655
	Medication	12,880 (1.0)	29 (2.2)	87 (1.8)	177 (1.7)	591 (1.4)	11,629 (1.0)	367 (1.2)	< 0.001
ASD	Yes	14,394 (0.7)	190 (7.6)	355 (4.2)	450 (2.5)	921 (1.2)	12,085 (0.6)	393 (0.8)	< 0.001
	Diagnosis age [Mean (S.D.)]	3.8 (2.2)	2.1 (2.1)	1.9 (2.2)	2.2 (2.2)	3.1 (2.3)	4.0 (2.1)	4.1 (2.0)	< 0.001
Total	2,143,652 (100)	2512 (100)	8401 (100)	18,220 (100)	75,627 (100)	1,988,053 (100)	50,839 (100)		

*ADHD* attention-deficit/hyperactivity disorder, *ASD* autism spectrum disorder, *S.D.* standard deviation.

^a^The proportion by birth weight group was calculated using the annual number of births as the denominator.

^b^The analysis of ADHD included children born between 2008 and 2010, taking the average age at ADHD diagnosis (n = 1,248,909) into account.

### ADHD

**Figure 2:**
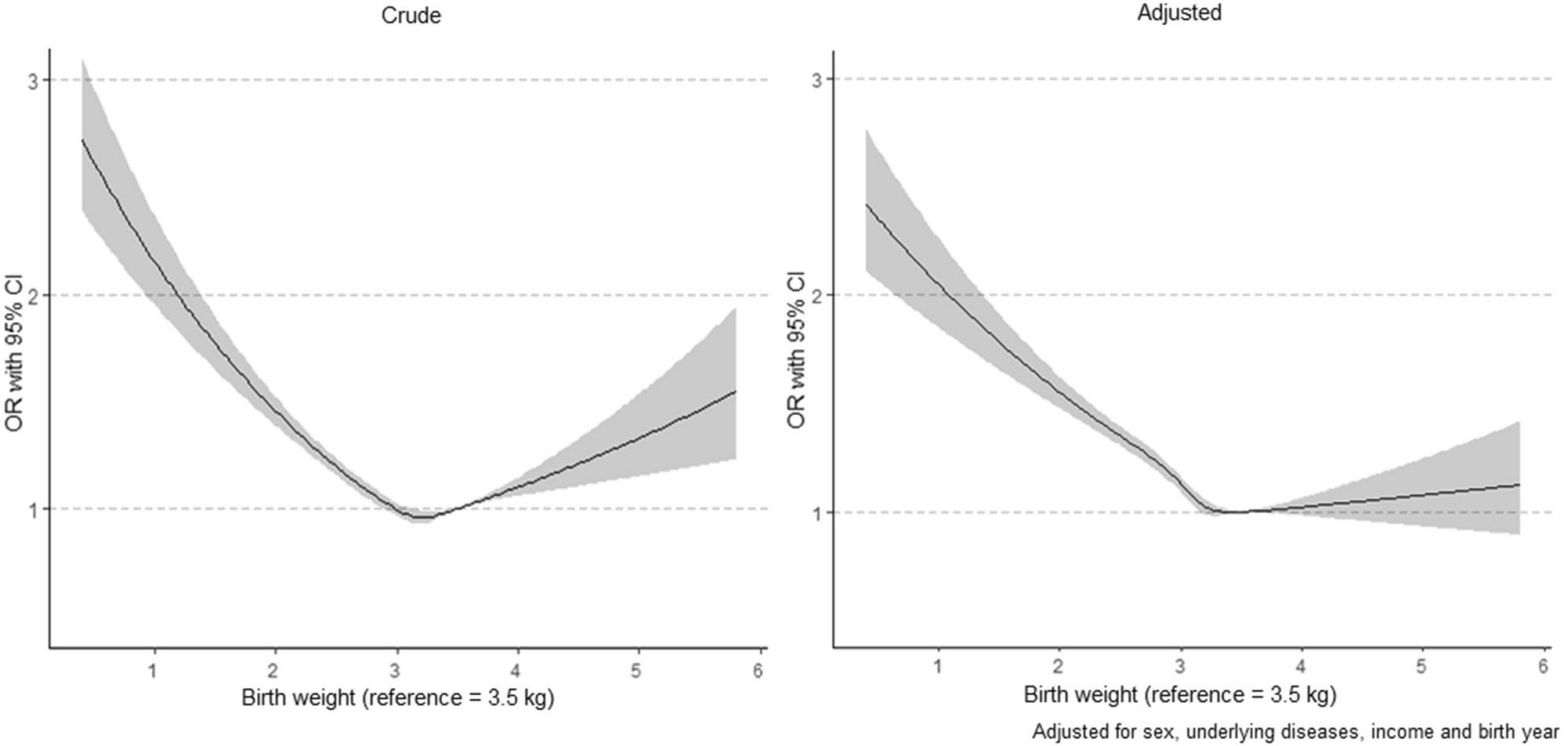
Crude and adjusted association between birth weight as a continuous variable expressed as cubic spline and ADHD.

Among children who were born between 2008 and 2010, 2.2% were diagnosed with ADHD (at approximately 7 years of age, regardless of birth weight) and approximately 50% were prescribed medication (Table 1). The LBW group showed a higher proportion of having ADHD. Approximately 4.7% of children with birth weights less than 1.0 kg were diagnosed with ADHD, but 2.1% of those in the NBW group were diagnosed with ADHD (Table 1). After adjustment for potential confounders, infants with birth weights of 2.0–2.4 kg and 1.5–1.9 kg had an adjusted odds ratio (OR) of being diagnosed with ADHD; OR 1.41 (95% confidence interval [CI] 1.33–1.50) and OR, 1.49 (95% CI 1.33–1.66), respectively, as compared with the NBW group (Table 2). Birth weights over 4.0 kg were associated with having ADHD, but were not statistically significant after adjusting for confounding factors. Additionally, we found similar associations when birth weight was studied as a continuous variable expressed as cubic splines. A stronger association between ADHD and birth weight was observed with decreasing birth weight (Fig. 2). A similar tendency was noted in the groups with and without medication (Supplementary Table S2). Subgroup analysis for infants who did not have a disease history also showed comparable results (Table 3).

**Table 2:** Associations of birth weight with ADHD and ASD.

Birth weight (kg)	ADHD (n = 1,248,909)^a^			ASD (n = 2,143,652)		
	n (%)	OR (95% CI)	aOR (95% CI)^b^	n (%)	OR (95% CI)	aOR (95% CI)^b^
**< 1**	63 (4.7)	2.28 (1.77–2.93)	2.24 (1.73–2.89)	190 (7.6)	13.37 (11.53–15.53)	10.57 (9.08–12.31)
**1–1.4**	186 (3.8)	1.81 (1.56–2.10)	1.69 (1.45–1.96)	355 (4.2)	7.21 (6.48–8.04)	5.51 (4.94–6.16)
**1.5–1.9**	346 (3.3)	1.60 (1.43–1.78)	1.49 (1.33–1.66)	450 (2.5)	4.14 (3.76–4.55)	3.25 (2.95–3.59)
**2.0–2.4**	1,219 (2.9)	1.36 (1.29–1.45)	1.41 (1.33–1.50)	921 (1.2)	2.02 (1.88–2.16)	1.91 (1.79–2.05)
**2.5–4.0**	24,412 (2.1)	Ref	Ref	12,085 (0.6)	Ref	Ref
** > 4.0**	782 (2.5)	1.20 (1.12–1.29)	1.03 (0.96–1.11)^c^	393 (0.8)	1.27 (1.15–1.41)	1.10 (1.00–1.12)^c^

*ADHD* attention-deficit/hyperactivity disorder, *ASD* autism spectrum disorder, *OR* odds ratio, *aOR* adjusted odds ratio, *CI* confidence interval.

^a^The analysis of ADHD included children born between 2008 and 2010, and the analysis of ASD included children born between 2008 and 2012.

^b^Adjusted for sex, history of congenital or perinatal diseases, income and birth year.

^c^*P* ≥ 0.05.

**Table 3:** Associations of birth weight with ADHD and ASD in children with no history of congenital or perinatal diseases.

Birth weight (kg)	ADHD (n = 950,673) ^a^			ASD (n = 1,623,972)		
	n (%)	OR (95% CI)	aOR (95% CI) ^b^	n (%)	OR (95% CI)	aOR (95% CI) ^b^
**1.5–1.9**	77 (2.8)	1.39 (1.11–1.74)	1.45 (1.16–1.83)	51 (1.1)	2.07 (1.58–2.73)	2.20 (1.67–2.90)
**2.0–2.4**	660 (2.6)	1.30 (1.20–1.41)	1.43 (1.32–1.55)	395 (0.9)	1.66 (1.50–1.84)	1.83 (1.65–2.03)
**2.5–4.0**	18,484 (2.0)	Ref	Ref	8378 (0.5)	Ref	Ref

*ADHD* attention-deficit/hyperactivity disorder, *ASD* autism spectrum disorder, *OR* odds ratio, *aOR* adjusted odds ratio, *CI* confidence interval.

^a^The analysis of ADHD included children born between 2008 and 2010, and the analysis of ASD included children born between 2008 and 2012.

^b^Adjusted for sex, income and birth year.

### ASD

**Figure 3:**
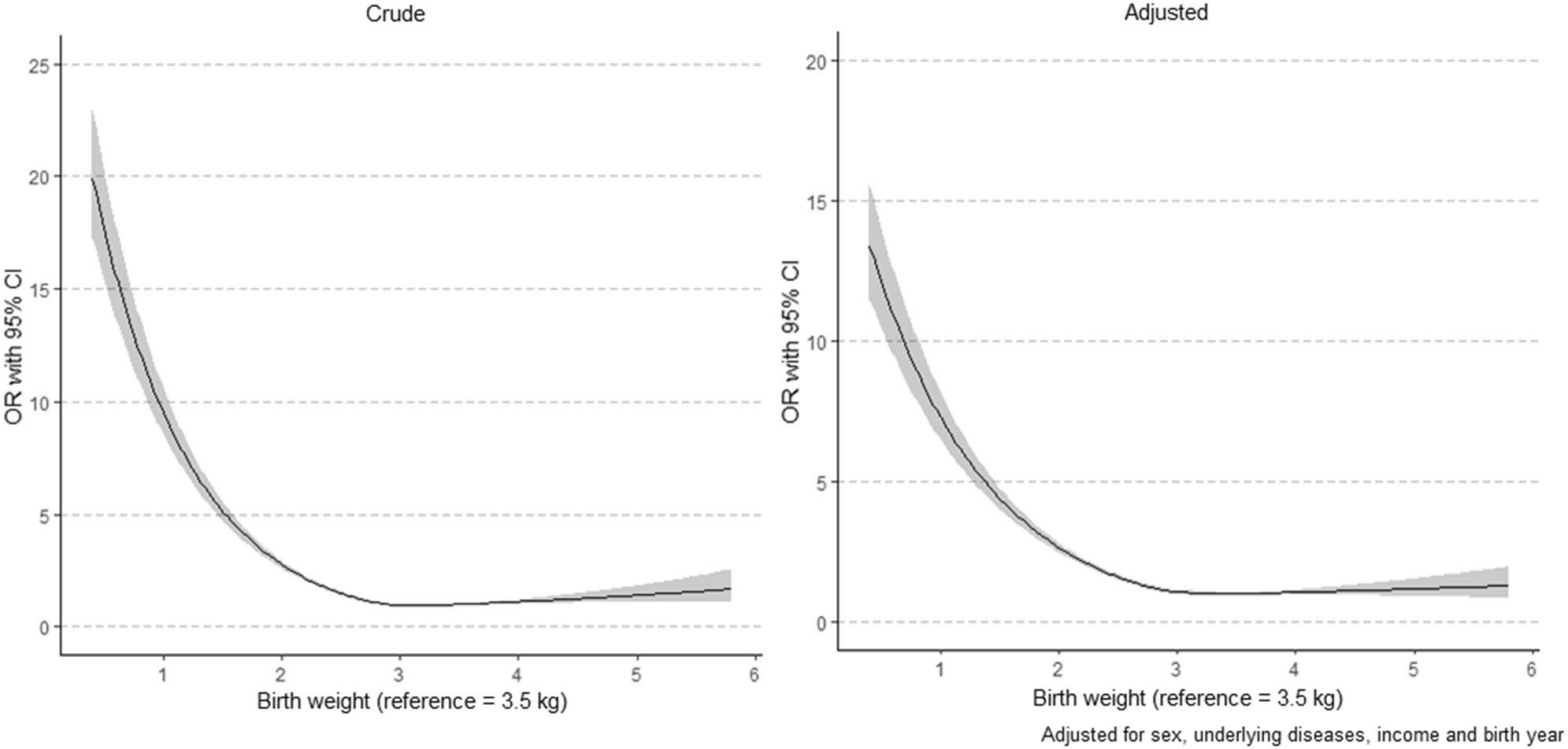
Crude and adjusted association between birth weight as a continuous variable expressed as cubic spline and ASD.

Approximately 0.7% of children who were born between 2008 and 2012 were diagnosed with ASD and LBW infants were more likely to be diagnosed with ASD earlier than were NBW infants (Table 1). Extremely low birth weight infants weighing less than 1 kg have an adjusted OR being diagnosed with ASD; OR,10.57 (95% CI 9.08–12.31) compared with NBW infants. Similarly, the OR in infants with birth weights of 2.0–2.4 kg and 1.5–1.9 kg was 1.91 (95% CI 1.79–2.05) 3.25 (95% CI 2.95–3.59), respectively, which were increased odds of having ASD (Table 2). Birth weights over 4.0 kg were associated with having ASD, but were not statistically significant in multivariable logistic regression. Additionally, similar associations were observed when birth weight was studied as a continuous variable (Fig. 3). Among children without congenital or perinatal diseases, those with birth weights of 1.5–1.9 kg and 2.0–2.4 kg had OR of 2.20 (95% CI 1.67–2.90) and 1.83 (95% CI 1.65–2.03), respectively, indicating increased odds of having ASD (Table 3).

## Discussion

This study assessed the association between birth weight and the diagnosis of ADHD and ASD using a large national cohort. VLBW infants and those with birth weights of 1.5–2.4 kg had increased odds of having ADHD and ASD, as compared with NBW. Birth weights over 4.0 kg were associated with having these disorders, but were not statistically significant after adjusting for confounding factors. We analyzed children without congenital or perinatal diseases, thereby controlling for disease history, and obtained higher odds in the lower birth weight groups.

A previous large national cohort study in Sweden reported that gestational age was independent of the covariates and familial factors for being diagnosed with ADHD and autism. However, the study had limited information about congenital or perinatal diseases^13^. In a Norwegian study of 903,402 infants who were born between 1967 and 1983, researchers reported a nonsignificant higher risk (relative risk, 0.8; 95% CI 0.4–1.4) of ASD among late preterm infants (gestational age 34–36 weeks). The study showed that late preterm infants had increased risks of developing psychological, behavioral, and emotional disorders but it was not limited to ADHD^11^. A previous case–control study conducted in Finland found that infants with LBW (1.5–2.5 kg) had an increased risk of autism (adjusted OR, 1.57; 95% CI 1.1–2.3) and pervasive developmental disorders (adjusted OR, 1.81; 1.4–2.4), but there was no association with Asperger syndrome (adjusted OR, 1.22; 0.9–1.7)^14^. In the current study, we found that infants with birth weights of 2.0–2.4 kg and 1.5–1.9 kg were associated with a higher OR of having ADHD (adjusted OR, 1.41 and 1.49) and ASD (adjusted OR, 1.91 and 3.25). The trends were similar in the subgroup analyses for children without congenital or perinatal diseases. Furthermore, infants with birth weights over 4.0 kg had increased risks of ADHD and ASD but were not statistically significant after adjusting for confounding factors. These results are consistent with previous findings that neurodevelopmental disorders regarding with perinatal complications or socioeconomic status regarding with the gestational overgrowth^15,16^.

Compared with ADHD, the OR according to birth weight was greater in ASD. The pathogenesis of ADHD and ASD are multifactorial and include genetic susceptibility and intrauterine or postnatal environment. Brain regions that play key roles are different in these disorders^5,11–14,17–19^. Among these regions, the prefrontal cortex is known to be important for regulating attention, behavior, and emotion and has been found to contribute to ADHD^20^. The prefrontal cortex is often called a “late developing” region of the brain. Prefrontal development is affected by prematurity and social environment, such as exposure to maltreatment or poverty during childhood that results in symptoms^21^. The amygdala is one of the most important brain regions involved in the pathogenesis of ASD. The amygdala experiences the earliest prenatal structural and functional growth in the brain. Therefore, the region is vulnerable to perinatal diseases or extrauterine environments that LBW infants may encounter during the early neonatal period^22–25^. The difference of impact of birth weight between ADHD and ASD in the present study can be attributed to the differences in the period of development of these brain regions.

This study showed that children with lower birth weight were diagnosed with ASD earlier. VLBW infants are usually encouraged to regularly visit outpatient clinics to monitor long-term follow-up outcomes. This routine appears to affect earlier diagnosis in the VLBW group. In contrast, infants with birth weights of greater than 2.0 kg are diagnosed with ASD 1–2 years later than the average age of diagnosis. They could be a relatively less investigated group than those with VLBW, and the risk of neurodevelopmental disorders in these children might have been overlooked. Previous studies have suggested that early intervention can improve the outcomes of children with ADHD and ASD^26,27^. Establishing a proper screening system is important to identify ADHD and ASD early and provide appropriate intervention ^28,29^. The American Academy of Pediatrics (AAP) recommends screening all children for ASD at 18 and 24 months of age during their primary care visits using a combination of general developmental screening tools and standardized autism-specific screening tests^30^. However, while the AAP does not recommend universal screening for ADHD, it recommends pediatricians or primary care clinicians to screen for ADHD when academic or behavioral problems and ADHD symptoms exist in children from 4 to 18 years of age^31^. In Korea, physicians probe for ASD with two or three questions during the National Health Screening Program for Infants and Children (NHSPIC); nevertheless, no validated universal ASD-specific screening program exists^32^. Introducing an ASD screening program suitable for the health system of each country is necessary, and establishing a universal ASD-specific program should be considered, at least for high-risk groups, such as those with LBW. As regards ADHD, it is important to inform the caregiver or educational institution of the ADHD symptoms so that a referral can be made.

The major strength of present study lies in the fact that more robust estimates could be made based on the nationwide live-born data from the NHIS database. This increases the generalizability of the results and may minimize bias. Furthermore, this study was not limited to a single birth weight category but assessed all newborns; hence, we could explore the possible association between neurodevelopmental outcomes and birth weight category. Previous studies have seldom analyzed birth weights other than 2.0–2.4 kg or greater than 4.0 kg. In addition, we analyzed two representative neurodevelopmental disorders and compared their association with birth weight. The results were controlled for congenital or perinatal diseases and income level of the family; furthermore, subgroup analysis was performed for children without associated diseases.

This study had some limitations. First, we did not have access to gestational age data, which was one of the most important prognostic factors, and this hindered us from assessing the associations of small for gestational age and neurodevelopmental disorders. The major reason is that the NHIS does not provide the gestational age in order to protect personal information. We considered using the codes from the International Classification of Diseases 10th revision (ICD-10) for preterm births, but the number of infants who had those codes was only half of the number of preterm infants in the Korean birth statistics. Moreover, the codes were divided into two or three groups based on the gestational age of 28 (until 2010) or 32 weeks (until 2015) in Korea. Therefore, it was impossible to assess the prognosis of late preterm infants who corresponded to the group of interest in this study with the codes. Until now, utilizing birth weight data in the NHSPIC has been the best method to access birth information in Korea, and constant efforts are required to change the policy such that the NHIS can provide more birth information. Second, we could not obtain data on the family’s medical history, such as maternal pregnancy complications or parents’ and siblings’ mental health history. With family history, it would be possible to assess hereditary aspects of neurodevelopmental disorders^33^. Establishing a national birth cohort that can link the family health data with the infants’ long-term outcomes could help assess and manage the health of high-risk infants. Finally, this study revealed the association between birth weight and neurodevelopmental disorders, but not the causality. However, the factors identified in this study may help identify the pathogenesis of these disorders.

Despite the improvements in neonatal care, LBW (especially 1.5–2.4 kg) infants born between 2008 and 2012 still had higher ADHD and ASD risk than those with NBW. In particular, children born with LBW without congenital or perinatal history had similar results. Physicians in tertiary hospitals as well as those in primary care clinics who often follow-up children born with LBW should be aware of this. Additionally, further studies on detailed clinical follow-up and screening for neurodevelopmental disorders of LBW are required.

## Methods

### Data source

Korea achieved universal health coverage in 1989, and all national health insurance and medical aid claims data for insurance-covered medical services that all medical institutions provide are managed in a single centralized database. This database contains information on demographics, healthcare utilization, expenditures, and diagnoses based on the ICD-10. Upon researchers request, the NHIS provide the integrated data with NHSPIC results: birth weight, weight, height, and developmental assessment at screening. The NHSPIC began in November 2007. The program targeted infants and children aged less than 6 years; their growth and development are monitored seven times overall^32^. By 2019, 94.6% of children underwent a check-up at least once in their lifetime^34^. During their check-ups, parents reported the children’s birth weight as indicated in the birth certificate issued by the birth medical institutions. A study introduced the NHIS-NHSPIC cohort with children born in 2008 and 2009, and we extended the cohort to children born in 2012^35^. Several studies were conducted using the Korean NHIS cohorts and recently, studies regarding long-term outcomes of newborns using the NHIS database were published^36–39^.

This study was designed and performed in accordance with the principles of the Declaration of Helsinki. The institutional review board (IRB) approval statement and the research plan summary were submitted to the NHIS website to gain access to the database. These were approved by the review committee that decides on the provision and use of national health information data to assigned research institutions^40^. The National Cancer Center IRB reviewed and exempted this study (NCC2019-0292) from the requirement of obtaining informed consent because the NHIS data are anonymized. We were granted access to all data of children born between 2008 and 2017.

### Study population

This study included all live births between 2008 and 2012; however, considering the average age at ADHD diagnosis, analyses for ADHD were performed for children born between 2008 and 2010, excluding live births from 2011 and 2012^41^. We excluded those who died before 2018 or those without the NHSPIC or income data. Moreover, we excluded those with birth weights less than 0.4 kg, considering that neonatal resuscitation was withheld for them, and assumed that the birth weight was recorded incorrectly^42^.

### Birth weight ascertainment

Birth weight was determined using NHSPIC data. Birth weight was categorized into six groups: < 1.0 kg, 1.0–1.4 kg, 1.5–1.9 kg, 2.0–2.4 kg, 2.5–4.0 kg (NBW), and > 4.0 kg.

### Outcome ascertainment

ADHD was diagnosed if the ICD-10 code F90 was reported twice in a child visiting the outpatient clinics or inpatient admission. We divided those with ADHD into two groups based on the presence or absence of prescription (including any history of being prescribed methylphenidate and/or atomoxetine). ASD cases were defined if the ICD-10 codes F84.0, F84.1, F84.5, F84.8, and F84.9 were reported at least twice. In addition, the age at diagnosis was identified using the date that the disease codes were first registered.

### Other study variables

Perinatal and socioeconomic factors associated with neurodevelopmental outcomes were identified using data from the NHIS database. Sex, history of congenital or perinatal diseases, and income level were used as adjustment variables. The type of insurance (national health insurance or medical aid) and the NHIS premium based on income levels were used as proxy indicators of income level. Medical aid beneficiaries were defined as the lowest income group. Furthermore, the NHIS group was divided into four categories: Category 1, lower 25% premium; Category 2, 25–50% premium; Category 3, 50–75% premium; and Category 4, upper 25% premium. A child was defined as having a history of congenital or perinatal disease if they were diagnosed with at least one of the following ICD-10 codes during the first year of life: congenital malformations, deformations, and chromosomal abnormalities (Q codes); metabolic disorders (E70–E90); or neonatal conditions (P10–P96).

Incidentally diagnosed and usually spontaneously regressing diseases, such as atrial septal defect (Q21.1) and patent ductus arteriosus (Q25.0), which are among the most prevalent 20 diseases in each category, were excluded to prevent over-adjustment (Supplementary Table S3).

### Statistical analysis

Pearson’s chi-square test and analysis of variance were used to identify demographic differences by birth weight. Logistic regression analysis was performed to assess the correlation between birth weight and neurodevelopmental disorders. The NBW group was the reference group for the analysis. Birth weight was also considered as a continuous variable expressed as cubic splines with five knots, which were divided into quintiles. Outcomes were adjusted for sex, history of congenital or perinatal diseases, income level, and birth year using multivariable logistic regression analysis. For ADHD, we performed a multinomial logistic regression analysis according to medication history. In addition, to reduce the impact of a history of congenital or perinatal diseases on birth weight, subgroup analysis was performed among subgroups of infants with birth weights of 1.5–4.0 kg that did not have any disease history. Over 80% of infants with birth weights under 1.5 kg were diagnosed with congenital or perinatal diseases; therefore, they were not included in this subgroup analysis. All analyses were performed using SAS (version 9.4; SAS Institute Inc., Cary, NC, USA) and R (version 4.03; R Foundation for Statistical Computing, IBM Corp., Armonk, NY, USA). *P* values of < 0.05 were considered significant, and ORs with 95% CIs were reported to elucidate the strengths of associations.

## Supplementary Information

Supplementary Information.

## Data Availability

De-identified individual participant data will not be made available.
