# Supplementary material for: Association between birth weight and neurodevelopmental disorders assessed using the Korean National Health Insurance Service claims data
Source: Sci Rep. 2022 Feb 8;12:2080. doi: 10.1038/s41598-022-06094-x (PMC8827104; doi:10.1038/s41598-022-06094-x)
Supplement: Supplementary file 1 — Supplementary Information. [file 41598_2022_6094_MOESM1_ESM.docx]

**Association between birth weight and neurodevelopmental disorders assessed using the Korean National Health Insurance Service claims data**

In Gyu Song^1^, Han-Suk Kim^2*^, Yoon-Min Cho^3^, You-na Lim^4^, Duk-Soo Moon^5^, Seung Han Shin^2^, Ee-Kyung Kim^2^, Joonsik Park^6^, Jeong Eun Shin^6^, Jungho Han^6^, Ho Seon Eun^6^

**Supplemental Information**

**Supplementary Table S1.** Comparison of demographic characteristics between subjects with complete data and those with incomplete data ^a^

|  |  | **Complete** | **Incomplete** | **p-value** |
| --- | --- | --- | --- | --- |
| Birth year | 2008 | 413 756 (19.3) | 55 492 (25.7) | <0.001 |
|  | 2009 | 403 475 (18.8) | 44 984 (20.9) |  |
|  | 2010 | 431 678 (20.1) | 42 757 (19.8) |  |
|  | 2011 | 438 828 (20.5) | 37 882 (17.6) |  |
|  | 2012 | 455 915 (21.3) | 34 557 (16.0) |  |
| Sex | Female | 1 040 430 (48.5) | 104 313 (48.4) | <0.135 |
| Congenital or perinatal diseases | Yes | 479 204 (22.4) | 43 439 (20.2) | <0.001 |
| ADHD ^b^ | Yes | 27 008 (2.2) | 2 303 (1.6) | <0.001 |
|  | Diagnosis age [mean (SD)] | 7.1 (1.6) | 7.1 (1.6) | 0.163 |
|  | Medication | 12 880 (1.0) | 1 113 (0.8) | <0.001 |
| ASD | Yes | 14 394 (0.7) | 1 550 (0.7) | <0.011 |
|  | Diagnosis age [mean (SD)] | 3.8 (2.2) | 3.6 (2.3) | <0.001 |
| Total | | 2 143 652 (100) | 215 672 (100) |  |

*ADHD* attention-deficit/hyperactivity disorder, *ASD* autism spectrum disorder.

^a^ Incomplete data were excluded from the current study.

^b^ The analysis of ADHD included children born between 2008 and 2010, taking the average age at ADHD diagnosis into account.

**Supplementary Table S2.** Associations of birth weight with ADHD analyzed according to the presence or absence of medication history

| **Birth weight (kg)** | **ADHD** | | | **ADHD without medication** | | | **ADHD with medication ^a^** | | |
| --- | --- | --- | --- | --- | --- | --- | --- | --- | --- |
|  | **n (%)** | **OR (95% CI)** | **aOR**  **(95% CI) ^b^** | **n (%)** | **OR (95% CI)** | **aOR**  **(95% CI) ^b^** | **n (%)** | **OR (95% CI)** | **aOR**  **(95% CI) ^b^** |
| **<1** | 63 (4.7) | 2.28  (1.77–2.93) | 2.24  (1.73–2.89) | 34 (2.5) | 2.35 (1.67–3.30) | 2.29 (1.63–3.23) | 29 (2.2) | 2.20 (1.52–3.18) | 2.18 (1.50–3.16) |
| **1–1.4** | 186 (3.8) | 1.81  (1.56–2.10) | 1.69  (1.45–1.96) | 99 (2.0) | 1.84 (1.51–2.25) | 1.71 (1.40–2.09) | 87 (1.8) | 1.78 (1.44–2.20) | 1.66 (1.34–2.06) |
| **1.5–1.9** | 346 (3.3) | 1.60  (1.43–1.78) | 1.49  (1.33–1.66) | 169 (1.6) | 1.49 (1.28–1.74) | 1.39 (1.19–1.62) | 177 (1.7) | 1.72 (1.48–1.99) | 1.61 (1.38–1.87) |
| **2.0–2.4** | 1,219 (2.9) | 1.36  (1.29–1.45) | 1.41  (1.33–1.50) | 628 (1.5) | 1.34 (1.24–1.45) | 1.39 (1.28–1.50) | 591 (1.4) | 1.34 (1.24–1.45) | 1.44 (1.32–1.56) |
| **2.5–4.0** | 24,412 (2.1) | Ref. | Ref. | 12,783 (1.1) | Ref. | Ref. | 11,629 (1.0) | Ref. | Ref. |
| **>4.0** | 782 (2.5) | 1.20  (1.12–1.29) | 1.03  (0.96–1.11) | 415 (1.3) | 1.22 (1.11–1.35) | 1.04 (0.95–1.15) | 367 (1.2) | 1.19 (1.07–1.32) | 1.02 (0.91–1.13) |

*ADHD* attention-deficit/hyperactivity disorder, *OR* odds ratio, *aOR* adjusted odds ratio, *CI* confidence interval.

^a^ Children who were prescribed methylphenidate or atomoxetine.

^b^ Adjusted for sex, history of congenital or perinatal diseases, income and birth year.

**Supplementary Table S3.** ICD-10 codes that were not included in congenital or perinatal diseases

| **ICD-10** | **Diseases** |
| --- | --- |
| E73 | Lactose intolerance |
| E86 | Volume depletion |
| E87 | Other disorders of fluid, electrolyte, and acid-base balance |
| P38 | Omphalitis of newborn |
| P51 | Umbilical hemorrhage of newborn |
| P59 | Neonatal jaundice from other and unspecified causes |
| P70 | Transitory disorders of carbohydrate metabolism specific to newborn |
| P71 | Transitory neonatal disorders of calcium and magnesium metabolism |
| P72 | Other transitory neonatal endocrine disorders |
| P74 | Other transitory neonatal electrolyte and metabolic disturbances |
| P83 | Other conditions of integument specific to newborn |
| P92 | Feeding problems of newborn |
| Q10 | Congenital malformations of the eyelid, lacrimal apparatus, and orbit |
| Q21.1 | Atrial septal defect |
| Q25.0 | Patent ductus arteriosus |
| Q38.1 | Ankyloglossia |

*ICD-10* International Classification of Diseases 10th revision
